# Management of late-term pregnancy in midwifery- and obstetrician-led care

**DOI:** 10.1186/s12884-019-2294-7

**Published:** 2019-05-22

**Authors:** Joep C. Kortekaas, Aafke Bruinsma, Judit K. J. Keulen, Frank P.H.A. Vandenbussche, Jeroen van Dillen, Esteriek de Miranda

**Affiliations:** 1 Department of Obstetrics and Gynaecology, Radboud University Medical Center, Geert Grooteplein Zuid 10, 6523 GA Nijmegen, the Netherlands; 2 Department of Obstetrics and Gynaecology, Amsterdam UMC, University of Amsterdam, Meibergdreef 9, 1105 AZ Amsterdam, Netherlands

**Keywords:** Late- term pregnancy, Postterm pregnancy, Midwifery-led care, Obstetrician-led care, Induction of labour, Management of care

## Abstract

**Abstract:**

Management of late-term pregnancy in midwifery- and obstetrician-led care.

**Background:**

Since there is no consensus regarding the optimal management in late-term pregnancies (≥41.0 weeks), we explored the variety of management strategies in late-term pregnancy in the Netherlands to identify the magnitude of this variety and the attitude towards late-term pregnancy.

**Methods:**

Two nationwide surveys amongst all midwifery practices (midwifery-led care) and all hospitals with an obstetric unit (obstetrician-led care) were performed with questions on timing, frequency and content of consultations/surveillance in late-term pregnancy and on timing of induction. Propositions about late-term pregnancy were assessed using Likert scale questions.

**Results:**

The response rate was 40% (203/511) in midwifery-led care and 92% (80/87) in obstetrician-led care. All obstetric units made regional protocols with their collaborating midwifery practices about management in late-term pregnancy. Most midwifery-led care practices (93%) refer low-risk women at least once for consultation in obstetrician-led care in late-term pregnancy. The content of consultations varies among hospitals. Membrane sweeping is performed more in midwifery-led care compared to obstetrician-led care (90% vs 31%, *p* < 0.001). Consultation at 41 weeks should be standard care according to 47% of midwifery-led care practices and 83% of obstetrician-led care units (*p* < 0.001). Induction of labour at 41.0 weeks is offered less often to women in midwifery-led care in comparison to obstetrician-led care (3% vs 21%, *p* < 0.001).

**Conclusions:**

Substantial practice variation exists within and between midwifery-and obstetrician-led care in the Netherlands regarding timing, frequency and content of antenatal monitoring in late-term pregnancy and timing of labour induction. An evidence based interdisciplinary guideline will contribute to a higher level of uniformity in the management in late- term pregnancies.

**Electronic supplementary material:**

The online version of this article (10.1186/s12884-019-2294-7) contains supplementary material, which is available to authorized users.

## Background

Maternity care in the Netherlands is provided by independent/ community midwives (midwifery-led care) and clinically working midwives and obstetricians (obstetrician-led care) [1–4]. Healthy women with an uncomplicated pregnancy and childbirth receive midwifery-led care. When complications arise or when an increased risk on adverse perinatal and or maternal outcomes during pregnancy or childbirth occur, women in midwifery-led care will be referred to obstetrician-led care for consultation/ surveillance and/ or for take over if necessary. The risk selection is performed by midwives in midwifery-led care in order to provide the most appropriate care for mother and foetus [5]. This risk selection is based on the ‘Obstetrical Indication List’ (VIL) and agreements of regional collaborations. The Obstetrical Indication List is a national Dutch evidence and/or consensus based document which contains nationwide agreements between midwives, gynaecologists and paediatricians on the required level of care for women with specific conditions or risk of complications [6]. Regional collaborations between midwifery-led care and obstetrician-led care are formalized in a ‘Maternity Care Network’ (MCN, in Dutch VSV), a regional partnership between an obstetric unit and all collaborating midwifery practices, in which local protocols are made based on national and international guidelines. Local protocols and agreements may differ between MCNs.

In the Netherlands in 2013, 167,159 women with an ongoing vital pregnancy ≥22 weeks were registered, 142,782 (85.4%) of them started pregnancy in midwifery-led care. 164,257 (98.3%) women had a singleton pregnancy, from which 152,323 (92.7%) gave birth at term. From this singleton term gestation group, 79,622 (52.3%) started labour in midwifery-led care and 45,335 (29.8%) gave birth in midwifery-led care, from which 26,175 (17.2%) at home [7].

Postterm pregnancy is defined as a pregnancy with a gestational age ≥ 42.0 weeks. Late-term pregnancy refers to a pregnancy between 41.0–41.6 weeks [8]. In the Netherlands in 2013, 2199 women (1.3%) gave birth postterm and 27,460 (16.7%) late-term, with an absolute risk of perinatal death of 0.23% in postterm pregnancies and 0.16% in late-term pregnancies [7]. There is no national guideline regarding management in uncomplicated late-term pregnancy. Obstetrical low risk women remain under the responsibility of midwifery-led care until 42.0 weeks. Between 41 and 42 weeks they can receive their antenatal checks in midwifery-led care or they are referred for consultations in obstetrician-led care. Internationally, women reaching late-term or postterm pregnancy are induced in order to reduce the risk on adverse perinatal or maternal outcomes [9–14]. However, there is no consensus regarding the optimal timing of induction of labour nor about the frequency and content of consultation(s) in late-term pregnancies. There is no clear evidence regarding performance of an ultrasound, determining amniotic fluid or assessing foetal growth in late-term pregnancy [9]. International guidelines and literature suggest that both an induction of labour during week 41 and expectant management until 42 weeks with or without any antenatal consultation(s)/surveillance could be considered [9, 12–16].

According to the ‘Obstetrical Indication List’, patients should be referred > 294 days of gestation (> 42.0 weeks) [6]. The Royal Dutch Organisation of Midwives (KNOV) representing midwifery-led care, recommends local interdisciplinary agreements regarding management in late-term pregnancy with the option of an antenatal consultation in obstetrician-led care between 41.0–41.6 weeks, to sweep membranes at 41.0 weeks and to refer to obstetrician-led care to plan an induction at 42.0 weeks [17, 18]. The Dutch Society for Obstetrics and Gynaecology (NVOG), representing obstetrician-led care, gives no details about the frequency and content of antenatal consultations at 41.0–41.6 weeks, but states that an induction of labour can be considered between 41.0–41.6 weeks on the patients request or at 42.0/42.1 and that this should be in accordance with local interdisciplinary agreements made in the MCN [15]. Due to the lack of evidence on how to monitor late-term pregnancy and the variety in local protocols there is wide practice variation in the management of late-term pregnancies.

In order to gain more insight in the magnitude of this practice variation in late- and postterm pregnancy in the Netherlands, two nationwide surveys were performed [19]. The first survey was performed in midwifery-led care at the end of 2011 – beginning of 2012 followed by a survey in obstetrician-led care in 2013 [19]. In between there was no change in the national guideline on postterm pregnancy, nor major publications regarding management or timing of induction in late-term pregnancy which could have influenced local policy. In this study we report results of both surveys.

## Methods

We performed a national survey amongst all 511 independent midwifery practices in the Netherlands to explore management of late-term pregnancy in midwifery-led care. Respondents were representatives on behalf of their practice. The Midwifery organisation KNOV mailed all Dutch midwifery practices that were known by them with their email address with a call to fill in the on-line survey. Furthermore a call was done twice by the KNOV in their two-weekly e-mail newsletter to their members. Subsequently, an email with a request to fill out a comparable survey was sent by the researchers to all 87 hospitals (42 non-teaching; 35 teaching and 10 academic hospitals) with an obstetric unit in 2013. This survey was based on the survey sent to midwifery practices. Both surveys included Yes/No questions, single answer multiple choice questions, open questions, and some clarifying questions depending on the answers given. Both surveys included questions concerning standard local policy in case of late-term pregnancy, frequency and content of antenatal consultations between 41 and 42 weeks, indications and timing of labour induction. A five-point Likert scale was used to evaluate level of agreement with six statements regarding management in late-term pregnancy (completely disagree – completely agree). Both surveys could be filled in anonymously, though the name of the hospital and the level of care were obligatory in the hospital survey. Ethical approval was deemed unnecessary according to Central Committee on Research Involving Human Subjects (CCMO), since it was a short survey without people being subjected to procedures or being required to follow rules of behaviour [20].

Data on timing, frequency and content of a late-term pregnancy consultation and data on management strategies is presented as counts (percentages) in both midwifery-led care and obstetrician led-care, comparisons are made using Chi-square or Fisher Exact tests, when appropriate. We used the Mann-Whitney U test to compare agreement with the propositions between midwifery-led care and obstetrician-led care. A two-sided *p*-value of < 0.05 was considered statistically significant. Statistical analysis was performed using SPSS version 23.0.

The Checklist for Reporting Results of Internet ESurveys (CHERRIES) is shown in Additional file 1.

## Results

In midwifery-led care, 40% (203/ 511) of the representatives of the midwifery practices responded to the survey in comparison to 92% (80/ 87) of the representatives of obstetric units in obstetrician-led care. Characteristics of respondents in both midwifery-led care and obstetrician-led care are shown in Table 1. Most midwifery practices (97%) are part of a MCN. Table 2 shows the origin of late-term pregnancy protocols used in midwifery-led care. Community midwives refer to two or more hospitals in late-term pregnancy in 56% (113/203) and in most cases 87% (98/113) these hospitals differ in their management in late-term pregnancy. In obstetrician-led care, most hospitals (93%) made their local late-term pregnancy protocols in cooperation with midwives from midwifery-led care, which is adhered to by midwives according to 76% (61/80) of respondents in obstetrician-led care (not shown in a Table).

**Table 1:** Responders in midwifery- and obstetrician-led care in comparison to national numbers

Level of care	Survey		National 2012^a^	
	n	%	n	%
Midwifery-led care practices	203	40%	511	100%
Group practice (3 midwives or more)	150	74%	313	61%
Duo practice	35	17%	114	22%
Solo practice	14	7%	84	16%
Other	4	2%	0	0%
Obstetrician-led care units	80	92%	87	100%
Non-teaching hospital	37	46%	42	48%
Teaching hospital	34	43%	35	40%
Academic teaching hospital	9	11%	10	11%

^a^ https://www.nivel.nl/en

**Table 2:** Origin of late-term pregnancy protocols in midwifery-led care

	n	%
Maternity Care Network (interdisciplinary)	97	48%
Local gynaecologists	39	19%
Obstetrical Indication List	34	17%
Own practice	11	5%
Regional agreement midwifery practices	10	5%
Other	12	6%
	203	100%

The regionally made protocols regulate the timing, frequency, content and policy of a late-term pregnancy consultation/surveillance in obstetrician-led care of low risk women. Table 3 shows the timing of the late-term pregnancy consultation in obstetrician-led care according to the respondents in both midwifery- and obstetrician led care which could be scheduled once, twice or ‘every other day’ depending on local agreements. The content of this late-term pregnancy consultation/surveillance is variable, but according to the representatives of obstetrician-led care, it is standard procedure during hospital consultation/surveillance to perform a foetal non-stress test/ cardiotocography (87%) and a transabdominal ultrasound (93%), in which amniotic fluid volume is determined (96%) and/or foetal biometry (40%). In obstetrician-led care, 21% stated that a vaginal examination is always performed and 72% performs membrane sweeping ‘on indication’. Membrane sweeping (if possible) is less often performed in obstetrician-led care 22/72 (31%) in comparison to midwifery-led care 184/203 (91%) (*p* < 0.001 RR: 0.34; 95% CI 0.24–0.48).

**Table 3:** Gestational age of late-term pregnancy consultation in obstetrician-led care according to respondents from both levels of care

	Midwifery-led care		Obstetrician-led care	
	n	%	n	%
≤41.0	22	11%	21	26%
41.1–41.3	103	51%	28	35%
41.4–41.6	51	25%	13	16%
≥42.0	12	6%	1	1%
no consultation	15	7%	0	0%
Other^a^	0	0%	17	21%
	203	100%	80	100%

^a^patients request, on indication, no exact timing

In midwifery-led care, there is a strong preference (77%) to refer to a hospital that does not induce labour at 41.0 weeks of gestation. Table 4 shows the management strategies between 41 and 42 weeks of low risk women stratified by level of care in late-term pregnancy according to obstetrician-led care. In obstetrician-led care, it is less often standard care to induce the women from midwifery-led care at 41.0–41.2 weeks in comparison to low-risk patients originating from obstetrician-led care (3% vs 21%; *p* < 0.001) and it is more often standard care to adhere to a policy of expectant management with consultations (56% vs 39%, *p* = 0.04). In obstetrician-led care, labour is induced from 41.0 weeks onwards when there is a maternal request for induction (88% always and 10% after counselling pros and cons for both management strategies) and always when foetal or maternal indications arise.

**Table 4:** Management strategies in late-term pregnancy between 41.0–42.0 weeks in obstetrician-led-care for low risk women referred from midwifery-led-care and for women primarily in obstetrician-led care

Management Strategies	Women referred from midwifery-led care		Women primarily in obstetrician-led care		*P*-value
	n	%	n	%	
IOL^b^ at GA^a^ 41.0–41.2	2	3%	17	21%	< 0.001^¥^
IOL^b^ at GA^a^ 41.3–41.5	1	1%	3	4%	0.62^¥^
EM^c^ with extra consultations in obstetrician-led care	45	56%	31	39%	0.03^§^
EM^c^ without extra consultations in obstetrician-led care	4	5%	3	4%	1^¥^
Individual based	11	14%	4	5%	0.10^¥^
Patients request	17	21%	22	28%	0.36^§^
	80	100%	80	100%	

¥ Fisher Exact

§ Chi square

^a^*GA* Gestational age

^b^*IOL* Induction of labour

^c^*EM* Expectant management until 42.0 weeks in the absence of foetal or maternal indications for induction of labour before 42 weeks and subsequent induction from 42 weeks onwards

Statements on management in late-term pregnancy were analysed between the professional groups. In both midwifery-led care and obstetrician-led care, there was no common agreement on the statements between the levels of care. Table 5 shows the percentage of agreement (agree and totally agree combined) for each proposition stratified by level of care. In midwifery-led care there was less agreement on the statement ‘a consultation in obstetrician-led care at 41 weeks should be standard practice’ (47% vs 83%) and ‘consultation in obstetrician-led care at 41 weeks reassures the midwife’ in comparison to obstetrician-led care (31% vs 50%). There were no significant differences in level of agreement on the propositions ‘consultation in obstetrician-led care at 41 weeks reassures women’ and ‘consultation in obstetrician-led care at 41 weeks reassures the gynaecologist’.

**Table 5:** Agreement (agree and strongly agree combined) on statements regarding obstetrician-led care consultations in late-term pregnancy according to level of care

	Midwifery-led care practices (*n* = 203)		Obstetrician-led care units (*n* = 80)		*P*-value*
	*n*	%	*n*	%	
Consultation at/in week 41 should be standard	96	47%	66	83%	< 0.001
Consultation at/in week 41 reassures pregnant women	119	59%	50	63%	0.62
Consultation at/in week 41 reassures midwife	63	31%	40	50%	< 0.001
Consultation at/in week 41 reassures gynaecologist	98	48%	48	60%	0.32

*Mann-Whitney U test based on Likert scale for agreement

## Discussion

### Main findings

In the Netherlands, individual and local protocols are made on management of care in women reaching 41.0 weeks of gestation because of the absence of an interdisciplinary national guideline regulating management of late-term pregnancy. Timing, frequency, content of antenatal monitoring in late-term pregnancy and timing of induction differs within and between obstetric caregivers in midwifery-led care and obstetrician-led care. Women in midwifery-led care receive more membrane sweeping in comparison to women in obstetrician-led care at 41.0 weeks. There is no consensus within and between midwifery-led care and obstetrician-led care regarding the optimal management in late-term pregnancy.

### Comparison to other studies

If a policy of expectant management until 42.0 weeks is chosen, regular antenatal checks in late-term pregnancy (41.0–42.0) are advised, though there is inconsistent evidence regarding the effectiveness of antenatal monitoring for the prevention of adverse perinatal outcome [9, 15, 18]. Studies are varying in timing, frequency and content of these antenatal checks [9, 10, 15, 21–23]. Some international guidelines advise to perform a transabdominal ultrasound measurement of amniotic fluid for timely detection of an oligohydramnios and to detect small-for-gestational age foetus [9, 15]. Determining foetal growth restriction by ultrasound at term is challenging but is regularly used in third trimester or prior to induction to detect foetal growth restriction [24–26]. There is conflicting evidence regarding the detection of small for gestational age (<p10) neonates when ultrasounds are performed in routine or on indication at term [27–29]. The DIGITAT-trial (*n* = 650), on induction or expectant management for term suspected intra uterine growth restriction, showed that most neonates in this study had a birth weight < p10, but still 26–34% were > p10 [29]. In the late-term period, a retrospective cohort study (*n* = 71,050) described the results of a comparison between a policy using a routine ultrasound examination (routine scan of biometry and amniotic fluid index) at 41 gestational weeks with a policy using ultrasound on clinical indication (e.g. suspicion on small for gestational age) at 41 weeks. A policy of routine screening lowered the incidence of an adverse perinatal outcome at term, probably due to timely detection of SGA-foetus [28].

According to a Cochrane systematic review on amniotic fluid assessment as screening test for preventing adverse pregnancy outcome, Nabhan et al. concluded that the use of the amniotic fluid index increases the rate of diagnosis of oligohydramnios and the rate of induction of labour without improvement in peripartum outcomes (NICU admissions, perinatal mortality, pH < 7,10, AS5’ < 7, presence of meconium, assisted vaginal delivery or caesarean section). Therefore, the single deepest vertical pocket measurement in the assessment of amniotic fluid volume during foetal surveillance is advised to use, whereby < 2 cm should be defined as oligohydramnios, though there has not been a systematic review on diagnostic accuracy of both assessments [27].

In a large Dutch trial (*n* = 742), sweeping the membranes in low risk women at 41 weeks decreased the risk of postterm pregnancy (23% vs 41% RR: 0.57, 95% CI 0.46–0.71) with a number needed to treat of 6 [17]. However, sweeping is not widely implemented in obstetrician-led care. According to both the KNOV and NVOG guidelines, sweeping the membranes is advised in late-term pregnancy in order to reduce the risk on a postterm pregnancy. We did not ask caregivers in obstetrician-led care why they do not routinely sweep the membranes in late-term pregnancy. This could be due to the usual ‘high risk’ population in obstetrician-led care in which termination of pregnancy is required before 41 weeks and cervical priming is a more certain method to start labour induction.

Postterm pregnancy (≥42.0 weeks of gestation) is regarded as a ‘high risk’ condition by the ‘Obstetrical Indication List’ (VIL). In late-term pregnancy; the Dutch NVOG recommends to grant a patients request to induce labour. The ACOG guideline recommends to consider induction of labour and the NICE guideline recommends to offer induction of labour between 41.0 and 42.0 weeks in accordance with patients preferences and local circumstances [9, 12, 15]. The recommendations to ‘consider’ or to ‘offer’ induction in late-term pregnancy may have contributed to the practice variation between maternity caregivers both in midwifery-led care and obstetrician-led care. Practice variation does not necessarily mean suboptimal care, especially when there are no high quality randomized controlled trials with adequate sample size or reviews available covering the required time frame of comparison. More results to support policy making in uncomplicated late-term pregnancies will be provided by two ongoing trials: the INDEX study (The Netherlands) and the SWEPIS study (Sweden), which both randomized low risk women at 41.0 weeks of gestation for induction of labour at 41.0 weeks or expectant management until 42.0 weeks [19, 30].

We showed that policy in late term pregnancy varies between and within midwifery-led care and obstetrician-led care. However, on patients level women’s voice plays an important role in the process of shared decision making. In obstetrician-led care the management strategy (induction or expectant management) in late- term pregnancy is solely based on women’s preference in ±20% while a request for induction of labour is nearly always respected by the caregiver in obstetrician-led care. This corresponds to the international guidelines stating that the decision of women should be respected, whether or not she wants labour to be induced [9, 10, 12, 15, 21, 22].

There are some differences in attitude within and between midwifery-led care and obstetrician-led care based on the percentage of agreement on the propositions on management in late-term pregnancy. In midwifery-led care, 47.5% agreed on ‘consultation at 41.0 weeks in obstetrician-led care should be standard’, in comparison to 82.5% in obstetrician led care. The need of a late-term pregnancy consultation/surveillance and its content, frequency and timing, is internationally discussed without supporting high levels of evidence [9, 10, 12, 15, 22].

### Strengths and limitations

Our study shows the results of the first two national questionnaires in maternity care on policy in late-term pregnancy. Because maternity care in the Netherlands is provided by both midwives and obstetricians, we combined the results of both questionnaires. The midwifery-led care questionnaire received a relatively low (203/511) response rate probably because the midwifery organisation did not had access to all email addresses of Dutch midwifery practices and the calls within the newsletter of the midwifery organisation are not very well read. However, coverage of postal codes of midwifery-practices shows that it represents a large proportion of the practices across the country. We received a good (90%) response rate from the representatives in obstetrician-led care. We did not send the questionnaire to all individual obstetric caregivers in midwifery- and obstetrician-led care but asked the views of the team because policy in the Netherlands is made within and with the teams. This leads to a representative overview of current practice in late-term pregnancy in the Netherlands. In the last 5 years, the national guidelines did not change during the study period, and the incidence of induction of labour in week 41 remained stable (30.6% in 2013 to 29.8% in 2016) [31, 32].

Both questionnaires were based on literature, (Dutch) guidelines and daily practice. Because both questionnaires contained also questions for the specific professional group, a selection was made of those questions which were asked in both survey’s. The questionnaires can be repeated after the implementation of a new interdisciplinary guideline, in order to compare possible differences in management strategy.

## Conclusion

In a high resource country as the Netherlands there is no consensus regarding the timing, frequency and content of consultations/surveillance in late-term pregnancy and on timing of labour within and between midwifery- and obstetrician-led care. Results of further studies are needed to develop an evidence based interdisciplinary guideline on management in late-term pregnancy which will contribute to a higher level of uniformity in the management in late- term pregnancies.

## Additional file

Additional file 1: SF 2 checklist for reporting result of internet Esurveys (CHERRIES) on both questionnaires. (DOCX 24 kb)

## Abbreviations

KNOV: The Royal Dutch Organisation of Midwives (Dutch: Koninklijke Nederlandse Organisatie van Verloskundigen)
MCN: Maternity Care Network (Dutch: VSV/Verloskundig Samenwerkings Verband)
NVOG: Dutch Society of Obstetrics and Gynaecology (Dutch: Nederlandse Vereniging voor Obstetrie & Gynaecologie)
VIL: Obstetrical Indication List (Dutch: Verloskundige Indicatie Lijst)

## References

[1] Offerhaus PM, Otten W, Boxem-Tiemessen JC, de Jonge A, van der Pal-de Bruin KM, Scheepers PL, Lagro-Janssen AL (2015). Variation in intrapartum referral rates in primary midwifery care in the Netherlands: a discrete choice experiment. Midwifery.

[2] de Jonge A, van der Goes BY, Ravelli AC, Amelink-Verburg MP, Mol BW, Nijhuis JG, Bennebroek Gravenhorst J, Buitendijk SE (2009). Perinatal mortality and morbidity in a nationwide cohort of 529,688 low-risk planned home and hospital births. BJOG : an international journal of obstetrics and gynaecology.

[3] Sandall J, Soltani H, Gates S, Shennan A, Devane D (2015). Midwife-led continuity models versus other models of care for childbearing women. *The Cochrane database of systematic reviews*.

[4] Evers AC, Brouwers HA, Hukkelhoven CW, Nikkels PG, Boon J, van Egmond-Linden A, Hillegersberg J, Snuif YS, Sterken-Hooisma S, Bruinse HW (2010). Perinatal mortality and severe morbidity in low and high risk term pregnancies in the Netherlands: prospective cohort study. BMJ.

[5] Amelink-Verburg MP, Buitendijk SE (2010). Pregnancy and labour in the Dutch maternity care system: what is normal? The role division between midwives and obstetricians. Journal of midwifery & women's health.

[6] Obstetrical Indication List (VIL),. Obstetrical Vademecum, guideline from the 'Committee of Midwifery' and 'Health Care Insurance Board'. 2003.

[7] Perinatal Registration Netherlands (Perined) (2012). Perined yearbook.

[8] FIGO: International Federation of Gynecology and Obstetrics, ICD-10 coding, O48. 2016. https://icd.who.int/browse10/2016/en#/O48.

[9] American College of Obstetricians Gynecologists (2014). Practice bulletin no. 146: management of late-term and postterm pregnancies. Obstet Gynecol.

[10] Department of Health Government of South Australia (2012). South Australian perinatal Practice guidelines: prolonged pregnancy.

[11] Mandruzzato G, Alfirevic Z, Chervenak F, Gruenebaum A, Heimstad R, Heinonen S, Levene M, Salvesen K, Saugstad O, Skupski D (2010). Guidelines for the management of postterm pregnancy. J Perinat Med.

[12] Vayssiere C, Haumonte JB, Chantry A, Coatleven F, Debord MP, Gomez C, Le Ray C, Lopez E, Salomon LJ, Senat MV (2013). Prolonged and post-term pregnancies: guidelines for clinical practice from the French College of Gynecologists and Obstetricians (CNGOF). Eur J Obstet Gynecol Reprod Biol.

[13] Wennerholm UB, Hagberg H, Brorsson B, Bergh C (2009). Induction of labor versus expectant management for post-date pregnancy: is there sufficient evidence for a change in clinical practice?. Acta Obstet Gynecol Scand.

[14] Gulmezoglu AM, Crowther CA, Middleton P, Heatley E (2012). Induction of labour for improving birth outcomes for women at or beyond term. Cochrane Data Syst Rev.

[15] Dutch Society of Obstetrics and Gynaecology (NVOG): Serotiniteit/ 'post-term pregnancy'. 2007.

[16] Hussain AA, Yakoob MY, Imdad A, Bhutta ZA (2011). Elective induction for pregnancies at or beyond 41 weeks of gestation and its impact on stillbirths: a systematic review with meta-analysis. BMC Public Health.

[17] de Miranda E, van der Bom JG, Bonsel GJ, Bleker OP, Rosendaal FR (2006). Membrane sweeping and prevention of post-term pregnancy in low-risk pregnancies: a randomised controlled trial. BJOG : an international journal of obstetrics and gynaecology.

[18] Royal Dutch Organisation of Midwives (2015). "Factsheet naderende serotiniteit (Laat-Terme zwagerschap)"/ fact-sheet impending postterm pregnancy (late-term pregnancy).

[19] Kortekaas JC, Bruinsma A, Keulen JK, van Dillen J, Oudijk MA, Zwart JJ, Bakker JJ, de Bont D, Nieuwenhuijze M, Offerhaus PM (2014). Effects of induction of labour versus expectant management in women with impending post-term pregnancies: the 41 week - 42 week dilemma. BMC Pregnancy Childbirth.

[20] Vragenlijstonderzoek (Dutch)/ Questionnaire Research [https://english.ccmo.nl/investigators/types-of-research/other-types-of-research/questionnaire-research].

[21] National Institute for Health and Care Excellence (NICE) (2015). Induction of labour overview.

[22] Clinical Practice Obstetrics C, Maternal Fetal Medicine C, Delaney M, Roggensack A, Leduc DC, Ballermann C, Biringer A, Delaney M, Dontigny L, Gleason TP, et al. Guidelines for the management of pregnancy at 41+0 to 42+0 weeks. J Obstet Gynaecol Can. 2008;30(9):800–23. Accessed 11 April 2019. 10.1016/S1701-2163(16)32945-0 18845050

[23] Haumonte JB, d'Ercole C (2011). Prolonged pregnancy: when should surveillance be started and what should be the frequency?. Journal de gynecologie, obstetrique et biologie de la reproduction.

[24] Sovio U, White IR, Dacey A, Pasupathy D, Smith GCS (2015). Screening for fetal growth restriction with universal third trimester ultrasonography in nulliparous women in the pregnancy outcome prediction (POP) study: a prospective cohort study. Lancet.

[25] Goetzinger KR, Odibo AO, Shanks AL, Roehl KA, Cahill AG (2014). Clinical accuracy of estimated fetal weight in term pregnancies in a teaching hospital. *The journal of maternal-fetal & neonatal medicine : the official journal of the European Association of Perinatal Medicine, the Federation of Asia and Oceania Perinatal Societies, the International Society of*. Perinatal Obstet.

[26] Peregrine E, O'Brien P, Jauniaux E (2007). Clinical and ultrasound estimation of birth weight prior to induction of labor at term. Ultrasound Obstet Gynecol.

[27] Nabhan AF, Abdelmoula YA. Amniotic fluid index versus single deepest vertical pocket as a screening test for preventing adverse pregnancy outcome. The Cochrane database of systematic reviews. 2008;(3):CD006593. 10.1002/14651858.CD006593.pub2 PMC6464731 18646160

[28] Lindqvist P, Pettersson K, Moren A, Kublickas M, Nordstrom L. Routine ultrasound examination at 41 weeks of gestation and risk of post-term severe adverse fetal outcome: a retrospective evaluation of two units, within the same hospital, with different guidelines. BJOG : an international journal of obstetrics and gynaecology. 2014. 10.1111/1471-0528.12654 24593288

[29] Boers KE, Vijgen SM, Bijlenga D, van der Post JA, Bekedam DJ, Kwee A, van der Salm PC, van Pampus MG, Spaanderman ME, de Boer K (2010). Induction versus expectant monitoring for intrauterine growth restriction at term: randomised equivalence trial (DIGITAT). BMJ.

[30] Elden H, Hagberg H, Wessberg A, Sengpiel V, Herbst A, Bullarbo M, Bergh C, Bolin K, Malbasic S, Saltvedt S (2016). Study protocol of SWEPIS a Swedish multicentre register based randomised controlled trial to compare induction of labour at 41 completed gestational weeks versus expectant management and induction at 42 completed gestational weeks. BMC Pregnancy Childbirth.

[31] Perinatal registration Netherlands (Perined) (2013). Perined yearbook.

[32] Perinatal Registration Netherlands (Perined) (2016). Perined yearbook.

