# Supplementary material for: Management of late-term pregnancy in midwifery- and obstetrician-led care
Source: BMC Pregnancy Childbirth. 2019 May 22;19:181. doi: 10.1186/s12884-019-2294-7 (PMC6532173; doi:10.1186/s12884-019-2294-7)
Supplement: Supplementary file 1 — SF 2 checklist for reporting result of internet Esurveys (CHERRIES) on both questionnaires. (DOCX 24 kb) [file 12884_2019_2294_MOESM1_ESM.docx]

Additional file 1: SF 2 checklist for reporting result of internet Esurveys (CHERRIES).

# Midwifery-led care:

| Design | Describe survey design | Obstetrical management was explored by inviting all midwifery practices in the Netherlands to fill out the questionnaire by a representative of the practice. This study was carried out by the use of an electronic survey. |
| --- | --- | --- |
| **IRB (Institutional Review Board) approval and informed consent process** | IRB approval | IRB approval was not needed for this anonymized survey among professionals. |
|  | Informed consent | The target population was informed on the purpose of the study (care in late-term pregnancy) by direct mail and in a directly mailed newsletter of the professional organization. When the link to the survey was opened, participants were informed that by filling out the questionnaire they will give approval for the anonymized use of the data. |
|  | Data protection | The following commercial web survey provider was used: Survey Monkey. All data were hosted by Survey Monkey. An e-link to the survey was created; the web survey provider did not dispose of the email addresses used for direct mailing. |
| Development and pre-testing | Development and testing | The questions were composed by two investigators. The web survey was tested in a small pilot in the target population before the start of the study. |
| **Recruitment process and description of the sample having access to the questionnaire** | Open survey versus closed survey | It was regarded as a closed survey (only for the target population of midwifery practices) though the survey could be opened without password |
|  | Contact mode | The target population was informed and invited by direct mail and in a newsletter which was directly mailed explaining the goals and purposes of the survey. This email included the link to the online survey. In order to enlarge the response rate, a second invitation was sent 2 weeks after the initial request by the same routes. |
|  | Advertising the survey | No advertising was used. |
| **Survey administration** | Web/E-mail | The link to the survey was included in an email. Respondents could only get access to the web based survey by clicking on the link. The data were automatically collected and stored by the survey provider after finishing. |
|  | Context | Not applicable. |
|  | Mandatory/voluntary | Responding to the survey was voluntary |
|  | Incentives | None |
|  | Time/Date | The survey was conducted in December 2011/January 2012. |
|  | Randomization of items or questionnaires | No items or questionnaires were randomized. |
|  | Adaptive questioning | Adaptive questioning was mostly used. Based on the answer, respondents were directed to an additional question or to the next question. |
|  | Number of Items | The survey consisted of 19 items. |
|  | Number of screens (pages) | Questions were shown in the same screen. |
|  | Completeness check | Most questions were multiple choice questions with the possibility to make a comment, though there were no mandatory questions. |
|  | Review step | Respondents were able to go back to change existing answers until the survey was finished or until they closed the survey. |
| **Response rates** | Unique site visitor | Responders were determined as an unique visitor by their IP address. |
|  | View rate (Ratio of unique survey visitors/unique site visitors) | Not applicable. We did not build in a tracking system. |
|  | Participation rate (Ratio of unique visitors who agreed to participate/unique first survey page visitors) | Not applicable. We did not build in a tracking system. |
|  | Completion rate (Ratio of users who finished the survey/users who agreed to participate) | The completion rate was 100% for 16 of the 19 questions. Three questions were skipped by 2 or 3 users. |
| **Preventing multiple entries from the same individual** | Cookies used | Cookies were used by the survey provider to recognize repeat users in order to prevent multiple answering |
|  | IP check | The IP address of the client computer was used to identify potential duplicate entries from the same user, The period of time that no two entries of the same IP address was allowed was 90 days. |
|  | Log file analysis | No log file analysis was performed. |
|  | Registration | Not applicable. |
| **Analysis** | Handling of incomplete questionnaires | A selection was made of relevant questions for the purpose of this article that were asked in both MLC and OLC survey. These questions had a 100% completion rate. |
|  | Questionnaires submitted with an atypical timestamp | Not applicable |
|  | Statistical correction | Not applicable. |

# Obstetrician-led care:

| Design | Describe survey design | This study was carried out by the use of an electronic survey. A link to the survey was sent in an e-mail by the researchers to the contact persons of obstetric units who are connected to the Dutch Consortium **for Healthcare Evaluation and Research in Obstetrics and Gynecology** or to the representatives of obstetric units in obstetrician-led care. |
| --- | --- | --- |
| **IRB (Institutional Review Board) approval and informed consent process** | IRB approval | IRB approval was not needed. It was obligatory to enter the name of the hospital. Entering the name was voluntarily and the survey was handled anonymized. |
|  | Informed consent | The participants were informed about the purpose of the study (exploration of care in late-term pregnancy) by direct mail. When the link to the survey was opened, participants were informed that by filling out the questionnaire they will give approval for the anonymised use of the data. |
|  | Data protection | The following commercial web survey provider was used: google.docs/forms. All data were hosted by google.docs. An e-link to the survey was created; the web survey provider did not dispose of email addresses. |
| Development and pre-testing | Development and testing | The questions were composed by three investigators. The web survey was tested in a small pilot study before the start of the study. |
| **Recruitment process and description of the sample having access to the questionnaire** | Open survey versus closed survey | It was regarded as a closed survey (only for the target population of obstetrician-led care) though the survey could be opened without password. |
|  | Contact mode | The target population received an email explaining the goals and purposes of the survey and asked for their participation. This email included the link to the online survey. In order to enlarge the response rate, a second and third invitation was sent and non-responding hospitals were called. |
|  | Advertising the survey | No advertising was used. |
| **Survey administration** | Web/E-mail | The link to the survey was provided in an email. Respondents could only get access to the web based survey by clicking on the link. The data were collected automatically after their submission. |
|  | Context | Not applicable. |
|  | Mandatory/voluntary | Responding to the survey was voluntary |
|  | Incentives | None |
|  | Time/Date | The survey was conducted in 2013 (May - October) |
|  | Randomization of items or questionnaires | No items or questionnaires were randomized. |
|  | Adaptive questioning | Adaptive questioning was mostly used. Based on the answer, respondents were directed to an additional question or to the next question. |
|  | Number of Items | The survey consisted of 27 items. |
|  | Number of screens (pages) | Questions on the same topic were combined on the same screen. All questions were preceded by an introducing text. After answering the questions, new questions were displayed on a new screen. |
|  | Completeness check | Most questions were multiple choice questions with the possibility to make a comment. Some questions contained an option to quantify a certain answer (eg. Amniotic fluid index in cm) As such, for every question an answer was needed from every participant. |
|  | Review step | Respondents were able to go back to change existing answers until the survey was finished or until they closed the survey. |
| **Response rates** | Unique site visitor | Responders were determined as an unique visitor by hospital and name (when provided) |
|  | View rate (Ratio of unique survey visitors/unique site visitors) | Not applicable. |
|  | Participation rate (Ratio of unique visitors who agreed to participate/unique first survey page visitors) | Not applicable |
|  | Completion rate (Ratio of users who finished the survey/users who agreed to participate) | All users completed the full survey. |
| **Preventing multiple entries from the same individual** | Cookies used | Cookies were used by the survey provider (google form) to recognize repeat users in order to prevent multiple answering |
|  | IP check | The IP address of the client computer was not used to identify potential duplicate entries from the same user. Duplicate database entries were based on name and were eliminated before analysis, with the most complete and, if more were complete, the first entry kept for analysis (n=8). |
|  | Log file analysis | No log file analysis was performed. |
|  | Registration | In order to complete the survey it was compulsory to fill in the name of the hospital and voluntarily to fill in the name of the respondent. |
| **Analysis** | Handling of incomplete questionnaires | Only completed surveys could be submitted. Therefore no incomplete surveys were included in the analysis. |
|  | Questionnaires submitted with an atypical timestamp | Not applicable. |
|  | Statistical correction | Not applicable. |
